# 

**Published:** 1898-08-06

**Authors:** 


**Notices of Births, Deaths, and Marriages** are inserted in this column at a charge of 2s. 6d. for an announcement not exceeding four lines.

### NOTICE TO CORRESPONDENTS.

All MS., Letters, Books for Review, and other matters intended for the Editor should be addressed The Editor, "The Hospital," The Hospital Building, 28 & 29, Southampton Street, Strand, W.C.

The Editor cannot undertake to return rejected MS., even when accompanied by stamped directed envelope.

**Notice to Advertisers and Subscribers.**

All Advertisements, Orders for Copies of The Hospital, and Business Communications should be addressed to **THE MANAGER, (Not to The Editor),** The Hospital, The Hospital Building, 28 & 29, Southampton Street, Strand, London, W.C.

The Cost of Subscription, post free, is as follows:—Per week, 2½d. per quarter, 2s. 8d.; per half-year, 5s. 3d.; per annum, 10s. 6d. Foreign:—Per annum, 15s. 2d.; payable, in all cases, in advance.

**NOTICE.—Vol. XXIII. of "The Hospital" (October, 1897, to March, 1898), in handsome cloth binding, is now on sale at the Office of this Journal, price 7s. 6d. Cases for binding the Numbers contained in this Volume, 1s. 6d. Index to Vol. XXIII., 2½d., post free.**

**The Monthly Part for August is now ready. Price 6d., by post 8d.**

### BOOKS RECEIVED.

The Scientific Press.

"Physiology, Experimental and Descriptive." By Buel P. Colton.

Andrew Reid and Co.

"University of Durham College of Medicine Calendar for the Year 1898-99."

John Heywood.

"Buxton: Its Baths and Climate." By Samuel Hyde, M.D.

S. Maw, Son, and Thompson.

"Catalogue of Patented Sanitary Appliances."

J. B Lippincott Co.

"Lippincott's Pocket Medical Dictionary." Edited by Ryland W. Greene, A.B.

Cassell and Co.

"Elements of Histology." By E. Klein, M.D., F.R.S., and J. S. Edkins, M.A., M.B.

**Periodicals, &c.**—Medical Press, Guyoscope, Charity Organisation Review, Literary Digest, Woman's Signal, English Illustrated Magazine, Newspaper Owner and Manager, Middlesex Hospital Journal, Sunday at Home, Public Health Journal, Scalpel, Guy's Hospital Gazette, Humanitarian, Leisure Hour, Boy's Own Paper, Leeds Hospital Magazine, St. Bartholomew's Hospital Journal.

### THE STUDENT OF MEDICINE.

### APPOINTMENTS.

F. W. Bailey, M.R.C.S.Eng., L.R.C.P.Lond., appointed Honorary Anæsthetist to the Royal Infirmary, Liverpool. J. B. Christopherson, M.A., M.D., B.C.Cantab., F.R.C.S.Eng., appointed Assistant Demonstrator of Anatomy at St. Bartholomew's Hospital, London. G. D. Collen, M.A., M.D., B.Ch., B.A.O., Dublin University, appointed Medical Officer for All Saints District of Northampton Union. W. Denby, M.B., C.M.Edin., appointed Medical Officer for the Fourth District of the Bradford Union. F. W. Dobbin, B.A.Dub., M.D., appointed Medical Officer of Health to the St. Albans Rural District Council. W. G. Dunwoody, M.D.Dub., appointed Medical Officer for the Downham District of the Ely Union. F. A. Elkins, M.D.Edin., appointed Medical Superintendent to the Leavesden Asylum.

